# Association of serum choline levels and all-cause mortality risk in adults with hypertension: a nested case–control study

**DOI:** 10.1186/s12986-021-00637-1

**Published:** 2021-12-20

**Authors:** Mengmeng Song, Benjamin P. Xu, Qiongyue Liang, Yaping Wei, Yun Song, Ping Chen, Ziyi Zhou, Nan Zhang, Qiangqiang He, Lishun Liu, Tong Liu, Kangping Zhang, Chunlei Hu, Binyan Wang, Xiping Xu, Hanping Shi

**Affiliations:** 1 Department of Gastrointestinal Surgery/Clinical Nutrition, Capital Medical University Affiliated Beijing Shijitan Hospital, Beijing, 100038 China; 2 Beijing International Science and Technology Cooperation Base for Cancer Metabolism and Nutrition, Beijing, 100038 China; 3 Department of Epidemiology and Department of Nutrition, Harvard TH Chan School of Public Health, Boston, MA 02115 USA; 4 State Key Laboratory of Natural Medicines, Research Center of Biostatistics and Computational Pharmacy, China Pharmaceutical University, Nanjing, 210009 China; 5 Key Laboratory of Precision Nutrition and Food Quality, Ministry of Education, Department of Nutrition and Health, Beijing Advanced Innovation Center for Food Nutrition and Human Health, College of Food Sciences and Nutritional Engineering, China Agricultural University, Beijing, 100083 China; 6 Institute for Biomedicine, Anhui Medical University, Hefei, China; 7 Shenzhen Evergreen Medical Institute, Shenzhen, China; 8 College of Pharmacy, Jinan University, Guangzhou, China; 9 Graduate School at Shenzhen, Tsinghua University, Shenzhen, China

**Keywords:** Hypertension, All-cause mortality, Serum choline, Nested case–control

## Abstract

**Background:**

Serum choline levels were associated with multiple chronic diseases. However, the association between serum choline and all-cause mortality in Chinese adults with hypertension remains unclear. The purpose of this study is to explore the association between serum choline concentrations and all-cause mortality risk in Chinese adults with hypertension, a high-risk population.

**Methods:**

A nested, case–control study was conducted that included 279 patients with all-cause death, and 279 matched, living controls, derived from the China Stroke Primary Prevention Trial (CSPPT). Baseline serum choline concentrations were measured by liquid chromatography with tandem quadrupole mass spectrometry (LC–MS/MS). Multivariate logistic regression analysis was used to assess the association of serum choline levels and all-cause mortality risk, with adjustment of pertinent covariables, including folic acid and homocysteine.

**Results:**

The median age of all participants was 64.13 years [interquartile range (IQR), 57.33–70.59 years]. The median serum choline concentration for cases (9.51 μg/mL) was higher than that in controls (7.80 μg/mL) (*P* = 0.009). When serum choline concentration was assessed as a continuous variable (per SD increased), there was a positive relation between serum choline levels and all-cause mortality risk [odds ratios (OR), 1.29; 95% confidence intervals (95%CI), 1.06–1.57; *P* = 0.010]. There was an increased all-cause mortality risk for participants in quartiles 2–4 (≥ 4.00 μg/mL; OR, 1.79; 95%CI, 1.15–2.78 compared with quartile 1 (< 4.00 μg/mL). In addition, non-drinking was found to promote the incidence of all-cause mortality for those with high choline concentrations.

**Conclusions:**

High serum choline concentrations were associated with increased all-cause mortality risk among Chinese adults with hypertension, compared to lower choline concentrations.

*Trial registration* clinicaltrials.gov Identifier: NCT007948885; UTL: https://clinicaltrials.gov/ct2/show/NCT00794885?term=NCT00794885&draw=2&rank=1.

## Introduction

All-cause mortality is one of the basic and most important indicators of a population’s health and is readily available from global and regional vital statistics. In an early report based on post hoc analyses of China Stroke Primary Prevention Trial (CSPPT) [1], baseline total homocysteine (tHcy) predicted a 5-year all-cause mortality independent of major lifestyle, and cardiovascular and metabolic risk factors. This study investigates the role of choline, another participant of one carbon metabolism, in all-cause mortality.

Choline is an essential micronutrient with a range of physiological functions in the body [2]. For example, choline serves as a precursor for phospholipids and acetylcholine and has been shown to affect neurodevelopment in rodents [3]. Choline is a major source of methyl donor to betaine [4], which is important for re-methylation of homocysteine to methionine as well as DNA and histone methylation. Choline has been associated with cancer [5], liver steatosis [6], cardiovascular disease and metabolic syndrome [7], however, the findings were inconsistent. Experimental studies reported that choline deficiency was associated with liver disease, atherosclerosis and possibly nervous system diseases [8], whereas choline or betaine supplementation ameliorated liver damage [9]. However, high choline intake was associated with increased cardiometabolic mortality in studies from three racially diverse populations [10]. In addition, studies have reported that high levels of plasma choline are associated with adverse cardiovascular risk factors [11]. Choline consumption may be protective for cancer [12], however, higher serum choline concentration was associated with increased risk of cancer [13]. Therefore, available data indicate that choline could be beneficial or harmful, depending on its levels and other covariables. It underscores the importance to identify optimal range of choline levels to maximize its health benefits and minimize undue harm.

Currently, epidemiologic evidence on the associations of choline with mortality remains limited and inconsistent. Two large US cohort studies revealed positive associations of choline intake with all-cause and CVD mortality [14], but a prospective study of Japanese adults showed no such associations [15]. Reversely, a large prospective cohort study revealed that higher serum choline levels at diagnosis were associated with better hepatocellular carcinoma survival outcomes [16]. Hence, in this large, prospective, follow-up study of the CSPPT, we performed a nested case–control study to assess the prospective association between the baseline serum choline concentrations and all-cause mortality risk in adults with hypertension, and further evaluated whether the associations were modified by sociodemographic and clinical characteristics and lifestyle factors.

## Material and methods

### Participants

The participants for this nested, case–control study were obtained from the China Stroke Primary Prevention Trial (CSPPT). The methods and major results of the CSPPT have been reported elsewhere [17]. In brief, the CSPPT was a multi-community, randomized, double-blind, controlled trial conducted from May 19, 2008 to August 24, 2013 in 32 communities in China. Eligible participants included men and women aged 45–75 years with hypertension. Hypertension was defined as: (1) seated, resting, systolic blood pressure (SBP) ≥ 140 mmHg; or (2) diastolic blood pressure (DBP) ≥ 90 mmHg; or (3) taking antihypertensive medication. The major exclusion criteria included history of physician-diagnosed stroke, myocardial infarction, heart failure, post-coronary revascularization, and/or congenital heart disease.

The CSPPT was approved by the Ethics Committee of the Institute of Biomedicine, Anhui Medical University, Hefei, China (FWA assurance number: FWA00001263) and registered with Clinical Trials.gov, NCT00794885. Written, informed consent was obtained from all participants prior to data collection.

### Intervention and endpoint events

In the CSPPT, a total of 20,702 eligible participants were randomly allocated, in a 1:1 ratio, to one of two different treatment groups: a daily oral dose of one tablet containing 10 mg enalapril only (the enalapril group); or a daily oral dose of one tablet containing 10 mg enalapril and 0.8 mg folic acid (the enalapril-folic acid group). All participants were followed up every 3 months where information on vital signs, study drug adherence, concomitant medication use, adverse events and possible endpoint events was collected and recorded by trained research staff and physicians.

Death events, a pre-specified endpoint of the CSPPT, was one of the second outcomes in this study. All cause death included death from any cause (cancer, stroke, etc.). Evidence of death was identified by hospital death certificates or home visit reports from investigators. All endpoint outcomes were assessed by the Endpoint Adjudication Committee of the study.

### Nested case–control study

**Fig. 1:**
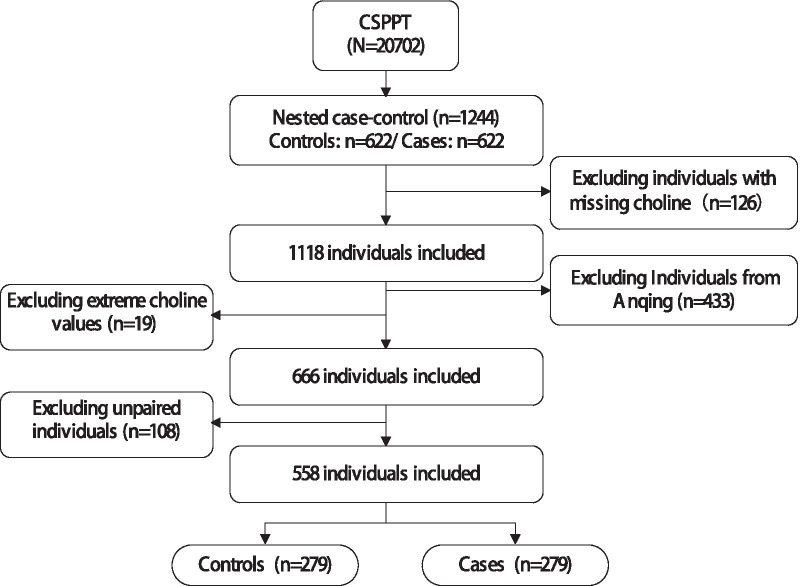
Flow chart of study participants

During a median treatment duration of 4.5 years (interquartile range, IQR: 4.2–4.7 years), a total of 622 participants died. A nested, case–control study was established that was derived from the 622 deceased patients who were matched with 622 living controls within this cohort. Controls were selected from those remaining participants who were still alive, and were matched by age (± 1 year), sex, treatment group (Enalapril and Enalapril-folic acid treatment) and study site with the cases in a 1:1 ratio. Although levels of serum choline vary according to the seasons of sampling, the significant difference can’t be explained by seasons only. Thus, we selected the participants from Lianyungang center (the majority of the population) in this current study. For the current analysis, 279 cases and 279 matched controls were included after excluding those with missing data and unpaired individuals (Fig. 1).

### Laboratory assays

At the baseline screening, morning serum samples were collected from all patients following an overnight fast. C677T gene (rs1801133) polymorphisms of 5,10-methylenetetrahydrofolate reductase (MTHFR), one of the genetic determinators of plasma homocysteine (HCY) levels, were detected with an ABI Prism 7900HT sequence detection system (Life Technologies) using the TaqMan assay. Serum HCY and creatinine were both measured using the cycling enzymatic method. Fasting blood glucose (FBG) was analyzed with the hexokinase/glucose-6-phosphate dehydrogenase method. Triglycerides (TG) and total cholesterol (TC) were both measured using the enzymatic colorimetric method. Serum high-density lipoprotein cholesterol (HDL-C) was measured using the direct test method, serum uric acid (SUA) was measured with the oxidase method, and serum folic acid was measured using a chemiluminescent immunoassay (New Industrial). Biochemical indexes were analyzed using automatic clinical analyzers (Beckman Coulter) at the central laboratory of the National Clinical Research Center for Kidney Disease, (Nanfang Hospital, Guangzhou, China). Plasma choline concentrations were measured by stable-isotope-dilution liquid chromatography-tandem mass spectrometry using 4500MD (AB SCIEX) in a commercial lab (Shenzhen Tailored Medical Laboratory, China).

### Statistical analysis

Continuous data were expressed as a median with interquartile range, and categorical data were expressed as n (%). Wilcoxon signed rank test were used to compare central estimates across ordered groups for non-normally distributed variables. Chi-square tests were used to compare proportions between groups.

Odds ratios (ORs) of all-cause mortality in relation to serum concentrations of choline were calculated using multivariate logistic regression models. Serum choline concentrations were categorized into quartiles based on its distribution. Multivariate logistic regression models were adjusted for age, body mass index (BMI), sex, month of blood sample collection, treatment group, smoking status, alcohol drinking, MTHFR C677T, systolic blood pressure, and triglyceride, cholesterol, folic acid, uric acid, glucose and homocysteine levels. In the stratified analysis, possible modifications of the association between serum choline as a categorical variable (high vs. low) and the all-cause mortality risks were assessed for variables including age (< 65, ≥ 65 years), sex, BMI (< 24.0 k/m^2^, ≥ 24.0 kg/m^2^), treatment group, smoking (yes, no), alcohol drinking (yes, no), MTHFR genotype (CC + CT, TT), homocysteine (< 13.72 μmol/L, ≥ 13.72 μmol/L), glucose (< 5.63 mmol/L, ≥ 5.63 mmol/L), total cholesterol (< 5.54 mmol/L, ≥ 5.54 mmol/L) and folic acid (< 6.498 ng/mL, ≥ 6.498 ng/mL) levels at baseline, using the median for cutoff points.

A two-tailed *P* < 0.05 was considered statistically significant in all analyses. R software (Version 4.0.4, http://www.R-project.org/) was used for all statistical analyses.

## Results

### Characteristics of the participants

This study included 279 cases of all-cause mortality during follow-up, and 279 matched living controls from the CSPPT cohort (Fig. 1). Among the 279 cases, 39 (14.0%) died of cancer and 10 (3.4%) from stroke. The median age of all participates was 64.13 years (IQR, 57.33–70.59 years). The median serum choline concentration was 8.99 μg/mL (IQR, 4.00–13.33 μg/mL). There were no major differences in baseline characteristics between cases and controls, other than cases had significantly higher choline concentrations (9.51 μg/mL vs. 7.80 μg/mL, *P* = 0.009) (Table 1).

**Table 1:** Baseline characteristics of cases and control

Variables	All patients (n = 558)	Controls (n = 279)	Cases (n = 279)	*P*
Age, years	64.13 (57.33–70.59)	64.13 (57.33–70.57)	64.12 (57.34–70.72)	0.976
Male, n (%)	318 (57.0)	159 (57.0)	159 (57.0)	1.000
BMI, kg/m^2^	24.54 (22.24–27.18)	25.04 (22.64–27.34)	24.34 (21.84–26.73)	0.033
Current smoking, n (%)	190 (34.1)	93 (33.3)	97 (34.8)	0.925
Current drinking, n (%)	181 (32.4)	96 (34.4)	85 (30.5)	0.551
Baseline SBP, mmHg	168.00 (156.00–181.83)	165.33 (154.67–180.67)	170.67 (157.33–183.00)	0.081
Baseline DBP, mmHg	94.67 (88.00–101.33)	93.33 (88.0–100.0)	96.67 (88.00–102.00)	0.152
Treatment group				
Enalapril	298 (53.4)	149 (53.4)	149 (53.4)	1.000
Enalapril-folic acid	260 (46.6)	130 (46.6)	130 (46.6)	
MTHFR C677T				
CC	137 (24.6)	70 (25.1)	67 (24.0)	0.626
CT	277 (49.6)	142 (50.9)	135 (48.4)	
TT	144 (25.8)	67 (24.0)	77 (27.6)	
Triglyceride, mmol/L	1.43 (1.06–1.99)	1.47 (1.05–2.09)	1.38 (1.07–1.93)	0.295
Total cholesterol, mmol/L	5.54 (4.78–6.22)	5.54 (4.94–6.21)	5.54 (4.64–6.22)	0.407
Uric acid, mg/dL	304.00 (256.00–356.00)	302.00 (254.50–354.00)	305.00 (257.00–361.00)	0.437
HDL cholesterol, mmol/L	1.29 (1.07–1.52)	1.29 (1.09–1.52)	1.29 (1.05–1.58)	0.967
Glucose, mmol/L	5.63 (5.17–6.34)	5.58 (5.17–6.32)	5.63 (5.19–6.40)	0.411
Creatinine, mmol/L	68.70 (58.30–79.28)	68.70 (57.35–80.05)	68.70 (58.55–76.95)	0.703
Serum Folic acid, ng/mL	6.50 (4.87–9.09)	6.57 (4.66–9.20)	6.50 (4.94–8.82)	0.915
Homocysteine, μmol/L	13.72 (11.02–18.82)	13.70 (10.78–18.27)	13.72 (11.17–19.25)	0.289
Choline, μg/ml	8.99 (4.00–13.33)	7.80 (3.37–12.72)	9.51 (4.45–13.89)	0.009

Continuous variables are presented as median (quantile1–quantile3), categorical variables are presented as n (%).

Differences in baseline characteristics between cases and controls were compared using χ^2^ tests for categorical variables and Wilcoxon signed rank tests for continuous variables.

SD, standard deviation; SBP, systolic blood pressure; DBP, diastolic blood pressure; MTHFR, 5,10-methylenetetrahydrofolate reductase; CC, CT and TT are different genotypes of MTHFR.

### Association of serum choline concentration with the all-cause mortality risk

**Fig. 2:**
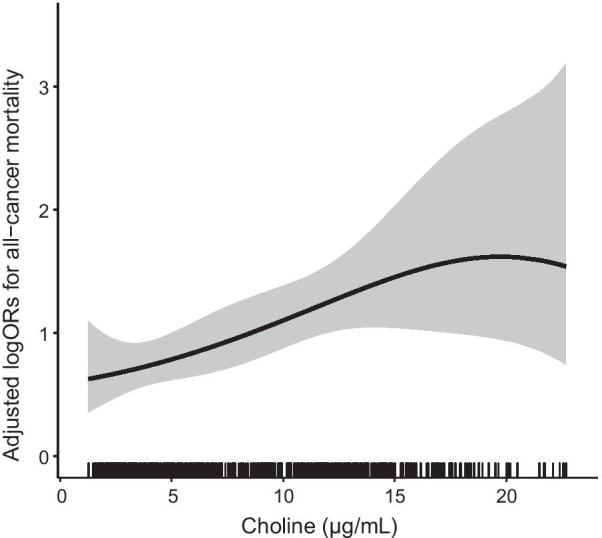
The association between baseline serum choline and all-cause mortality risk of participants. The splines were adjusted for body mass index, month of collecting blood samples, smoking, alcohol drinking, MTHFR C677T, systolic blood pressure, triglyceride, cholesterol, uric acid, folic acid, baseline fasting blood glucose, homocysteine and the matching factors (age, sex, treatment group)

The restricted cubic spline graph shows a positive association between serum choline concentration and all-cause mortality risk (Fig. 2). Overall, the all-cause mortality risk increased with the increase of serum choline concentration in hypertensive adults.

Consistently, when serum choline concentration was assessed as a continuous variable, a risk of mortality was found (OR, 1.29; 95%CI: 1.06–1.57, *P* = 0.010) with each SD increase of choline (per SD increase). When serum choline concentration was classified as quartiles, a significantly higher risk of all-cause mortality was found in participants in quartile 4 (≥ 13.33 μg/mL, OR, 2.40; 95% CI: 1.37–4.20; *P* = 0.002) compared with participants in quartile 1 (< 4.00 μg/mL). When quartiles 2 to 4 were combined together, a significant increased risk of all-cause mortality was found when compared to quartile 1 (OR, 1.79; 95%CI, 1.15–2.78, *P* = 0.010) (Table 2).

**Table 2:** The association between baseline serum choline and all-cause mortality risk

Choline, μg/mL	Cases/controls	Crude model		Adjusted model	
		OR (95%CI)	*P*	OR (95%CI)	*P*
Per SD increased	279/279	1.20 (1.02,1.41)	0.026	1.29(1.06–1.57)	0.010
Quartiles					
Quartile1 (< 4.00)	58/82	Reference		Reference	
Quartile2 (4.00–8.99)	71/68	1.48 (0.92–2.37)	0.107	1.59 (0.94–2.70)	0.084
Quartile3 (8.99–13.33)	70/69	1.43 (0.89–2.30)	0.135	1.59 (0.92–2.73)	0.094
Quartile4 (≥ 13.33)	80/60	1.89 (1.17–3.03)	0.009	2.40 (1.37–4.20)	0.002
*P* for trend			0.010		0.005
Categories					
Quartile1(< 4.00)	58/82	Reference		Reference	
Quartile2-Quartile4 (≥ 4.00)	221/197	1.59 (1.08–2.34)	0.020	1.79 (1.15–2.78)	0.010

Adjusted for age, body mass index, sex, month of collecting blood samples, treatment group, smoking status, alcohol drinking, MTHFR C677T, systolic blood pressure, triglyceride, cholesterol, folic acid, uric acid, glucose, homocysteine

### Stratified analyses by potential effect modifiers

**Fig. 3:**
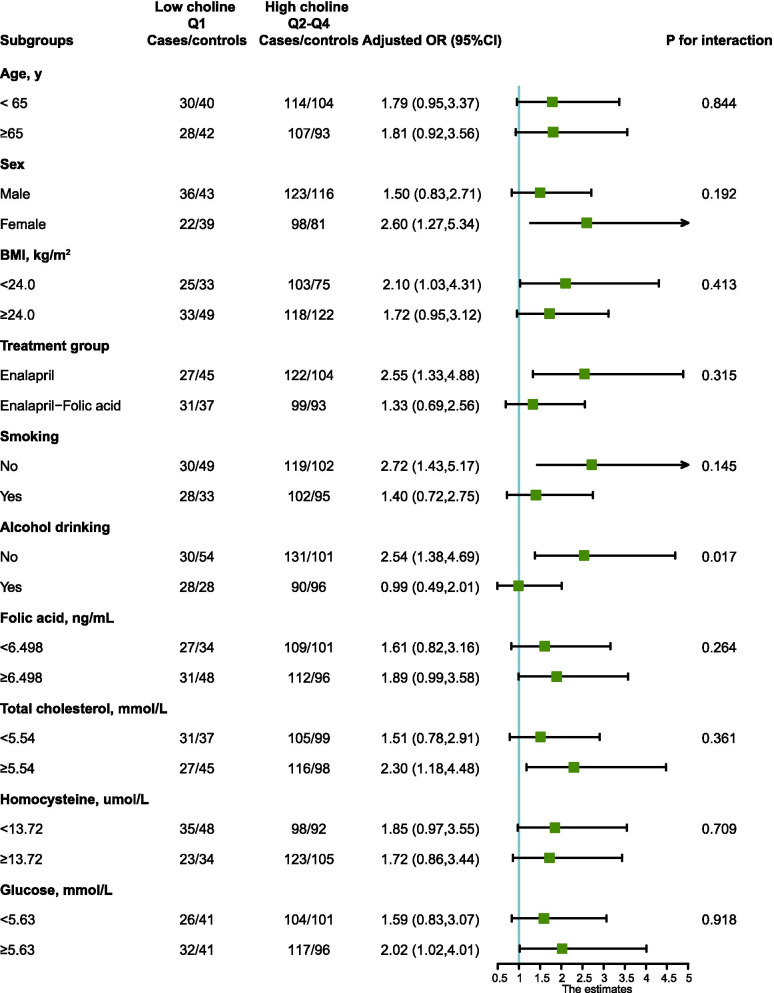
The association between serum choline concentrations and all-cause mortality risk in various subgroups. The association between serum choline and all-cause mortality risk in various subgroups in the nested case–control study within the China Stroke Primary Prevention Trial, conducted between May 2008 and August 2013. Adjusted for age, BMI, sex, treatment group, smoking status, alcohol drinking, MTHFR C677T, systolic blood pressure, triglyceride, cholesterol, uric acid, glucose, homocysteine, folic acid levels and month of collecting blood samples, if not stratified. OR, odds ratio. 95%CI, 95% confidence interval

Stratified analyses were performed to assess the association between serum choline concentrations and all-cause mortality risk in various subgroups (Fig. 3).

**Fig. 4:**
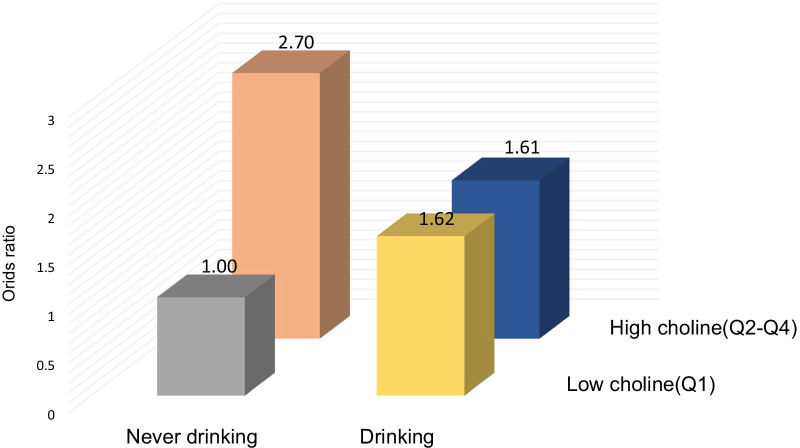
Interaction effect associations of baseline serum choline and alcohol with all-cause mortality risk

For those with increased choline concentrations, an increased risk of all-cause mortality was associated with female, treatment with enalapril, low BMI (< 24 kg/m^2^), enalapril treatment group, no drinking, no smoking, high glucose (≥ 5.63 mmol/L) and high total cholesterol (≥ 5.54 mmol/L). In addition, no-drinking were found to increase the all-cause mortality risk of patients with high serum choline concentrations, along with a significant interaction effect (*P* for interaction = 0.017). The visual interaction effect of drinking and serum choline on the all-cause mortality risk was shown in Fig. 4.

## Discussion

In this nested, case–control study which was derived from a prospective follow-up of the CSPPT, we found that among adults with hypertension, high serum choline concentrations (≥ 4.00 μg/mL) were associated with an increased risk of all-cause mortality compared to those with low serum choline levels (< 4.00 μg/mL, quartile 1). High levels (≥ 4.00 μg/mL) of choline in combination with being female, enalapril treatment, low BMI, never-drinking, never-smoking, high glucose or high total cholesterol were associated with a higher risk of all-cause mortality in adults with hypertension, and a significant interaction effect was found in those who were non-alcohol drinkers with a high serum choline concentration.

Choline is an essential nutrient for the normal functioning of cell membranes and muscle function, cholinergic neurotransmission, lipid transport and one-carbon metabolism [18]. Imbalanced choline intake can lead to fatty liver development, muscle damage [8], cardiovascular disease [19], cancer [20], cognitive decline [21] and osteoporosis [22]. In addition, the association between high choline intake/plasma choline level and mortality risk is controversial [15, 23, 24]. However, most previous studies focused on choline intake and cardiovascular disease mortality risk rather than serum choline and all-cause mortality. In addition, a meta-analysis showed that elevated concentrations of choline were associated with an increased risk of major adverse cardiac events (MACE, including death, myocardial infraction, stroke) independently of traditional risk factors [24]. However, the main outcomes of this meta-analysis included events other than death. Our study is the first to focus on the association of serum choline concentration and all-cause mortality in participants with hypertension, and found that high serum choline was associated with increased risk of all-cause mortality.

Cancer and cardiovascular disease (CVD) represent the leading cause of death and morbidity worldwide. Among the cases in our study, 14.0% and 3.4% developed cancer and stroke, respectively, during follow-up. A history of diabetes or CVD strengthened the choline-mortality association, as shown in many previous studies [14, 25, 26]. The participants in our study had hypertension, a risk factor of CVD, which may have strengthened the association between choline and mortality. In addition, those participants with both high serum choline and high total cholesterol had a higher risk of mortality, a finding that is consistent with previous studies, where it has been shown that clinical-based risks of CVD increased the choline-mortality association [27]. Another study showed that higher concentrations of plasma choline were associated with an unfavorable cardiometabolic risk-factor profile, including higher body mass index (BMI) [32]. Our study did not find the significant difference of BMI between participant with different serum choline level, probably because this study population was the adults with hypertension, with an overall high BMI.

Interestingly, we observed a trend of an inverse association of choline intake with all-cause mortality among alcohol drinkers or smokers. Given that choline is an important factor in maintaining liver function and that the vast majority of choline metabolism occurs in the liver [28], it is probable that in times of choline deprivation, varying degrees of liver damage and liver diseases may develop [29, 30]. As we all known, alcohol consumption was a main factor of liver function damage. High serum choline levels reflect high levels of choline metabolism, and a high choline level may help recover liver damage from excessive alcohol drinking and, in turn, lead to a phenomenon of reduced all-cause mortality risk happened in alcohol drinkers with high serum choline level. We had no relative data on liver function biomarkers in our study, but we observed an obvious inverse association of serum choline with deaths from all-cause among drinkers. In future studies investigating the health effects of choline-related nutrients, it will be important to take a patient’s cardiometabolic disease status and alcohol drinking status into consideration. In addition, a previous study reported that smoking decreases serum choline levels [31], which may be the cause the inverse phenomenon observed in our study.

However, it seems that the serum choline concentrations in our study are higher than those reported in previous studies [32, 33]. We explored four possibilities that may affect serum choline concentrations: laboratory measurement, study population of hypertension, the crosstalk of choline and folic acid., and sample storage length. Firstly, we worked with the lab that measured choline for this study to examine the purity of standard, detailed monitoring parameters, linearity curve, and reproducibility, and all of which met the quality control standards, indicating that the choline measurements for this study were accurate and reproducible. The second possibility is related to the study population with hypertension. This study was conducted in a hypertensive population with a mean age of 64 years. A recent choline study in Chinese population with a mean age of 58 years reported higher choline level in hypertensive participants compared to non-hypertensive controls [34]. Thus, our observed higher choline levels could be due to the fact that all of the study participants in our study had hypertension. This possible link between choline levels and hypertension remains to be verified by additional studies. The third possibility is related to low folate status in the study population. Choline participates in one carbon metabolic cycle, including sharing methylation pathways with folate. One study showed that choline and folate is metabolically inter-related; and choline is utilized as a methyl donor when folate intake is low [35]. As we published previously (17), the baseline serum folate level in the parent study population (CSPPT) is much lower than Western populations such as US population, due to the fact that China has not implemented mandatory folic acid grain fortification and folate intake in typical Chinese diet is low. In our study sample, baseline folic acid level was inversely correlated with choline levels. The complex interaction of choline and folate in patients with hypertension and the underlying mechanism is unclear. We hypothesize that higher serum level of choline may be a marker of low folate status, which is common in the Chinese patients with hypertension. The human body may attempt to absorb more choline from diets to compensate for the low folate status. This hypothesis needs to be explored and verified in the future study. The fourth possibility is related to samples storage and thaw cycle. Our study’s serum samples were stored in -80℃ freezer for about 10 years and underwent several freezing–thawing cycles. It is unclear to what extent the long-term storage and freezing–thawing cycles could impact choline levels. This again raises questions for future studies to consider, when using long-term stored serum samples, or when comparing findings across studies.

Our current study is novel for it uncovers the association of serum choline levels, a component of one-carbon metabolism, and the risk of all-cause mortality in an adult population with hypertension. The study results indicate that attention should be given to monitoring serum choline concentrations of patients with hypertension. Additionally, its strengths lie in the fact that this nested, case–control study was derived from a large, prospective cohort study, thereby avoiding recall bias. Participant serum choline concentrations were obtained from baseline blood samples prior to death, eliminating inverse causality.

There were also several limitations to this study. First, only baseline serum choline levels of the study participants were obtained. Repeated measurements of serum choline would have provided more information about all-cause mortality risk in relation to change in or cumulative serum choline levels. Second, this study was conducted in adults with hypertension, a high-risk population for cardiovascular morbidity and mortality; the extrapolation of these findings to populations without hypertension remains to be determined. However, adjustments for blood pressure measurements at baseline did not substantially change the findings. Third, this study represents a preliminary exploration of the association between choline and all-cause mortality risk. Our findings warrant further investigation, including simultaneously considering other components of one carbon metabolism.

## Conclusion

In this adult population with hypertension, we conducted the first prospective evaluation on the independent effect of serum choline concentrations on the risk of all-cause mortality. High serum choline levels were associated with an increased risk of all-cause mortality. These results are of potential scientific and clinical significance for death monitoring and control in adults with hypertension. Further studies are needed to confirm these findings in other populations.

## Data Availability

Data described in the manuscript, code book, and analytic code will be made available upon request after the review and approval of the institutional review board.

## Abbreviations

CSPPT: China stroke primary prevention trial
LC–MS/MS: Liquid chromatography with tandem quadrupole mass spectrometry
IQR: Interquartile range
OR: Odds ratios
95%CI: 95% Confidence intervals
total tHcy: Homocysteine
SBP: Systolic blood pressure
DBP: Diastolic blood pressure
MTHFR: Polymorphisms of 5,10-methylenetetrahydrofolate reductase
TG: Triglycerides
TC: Total cholesterol
HDL-C: High-density lipoprotein cholesterol
BMI: Body Mass Index
CVD: Cardiovascular disease
